# A case of herpes zoster ophthalmicus after third dose of Comirnaty (BNT162b2 mRNA) vaccine

**DOI:** 10.1111/dth.15411

**Published:** 2022-03-04

**Authors:** Fabrizio Martora, Gabriella Fabbrocini, Vincenzo Picone

**Affiliations:** ^1^ Dermatology Unit, Department of Clinical Medicine and Surgery University of Naples Federico II Naples Italy

Dear Editor,

The SARS‐CoV‐2 pandemic has afflicted the world in these 2 years. In Italy, the vaccination campaign has now reached the third dose. The third dose was initially directed to immunocompromised patients, but now it can be administered to patients who have received the second dose for at least 4 months.^1^ The side effects related to the administration of vaccines most described in the literature are fever, redness, pain and tenderness at the injection site, musculoskeletal pain and headache.^2^ Several cutaneous adverse events after anti‐COVID‐19 vaccination are described in the literature, such as delayed large local reaction, urticaria, morbilliform eruptions, herpes zoster, “COVID arm,” pityriasis rosea.^3^, ^4^, ^5^ Here, we report a case of a patient who presented with herpes zoster ophthalmicus (HZO) after the third dose of the Comirnaty (BNT162b2 mRNA) vaccine (Pfizer).

A 72‐years‐old female presented to our dermatology department with a vesicular eruption with diffuse erythema and dermatomeral distribution localized to the left side of the face (Figure 1). During history collection, hypertension, well controlled with drug therapy, was the only reported comorbidity. No acute or chronic onset disease events were identified during the collection of the history that could be related to the dermatologic manifestations.

**FIGURE 1:**
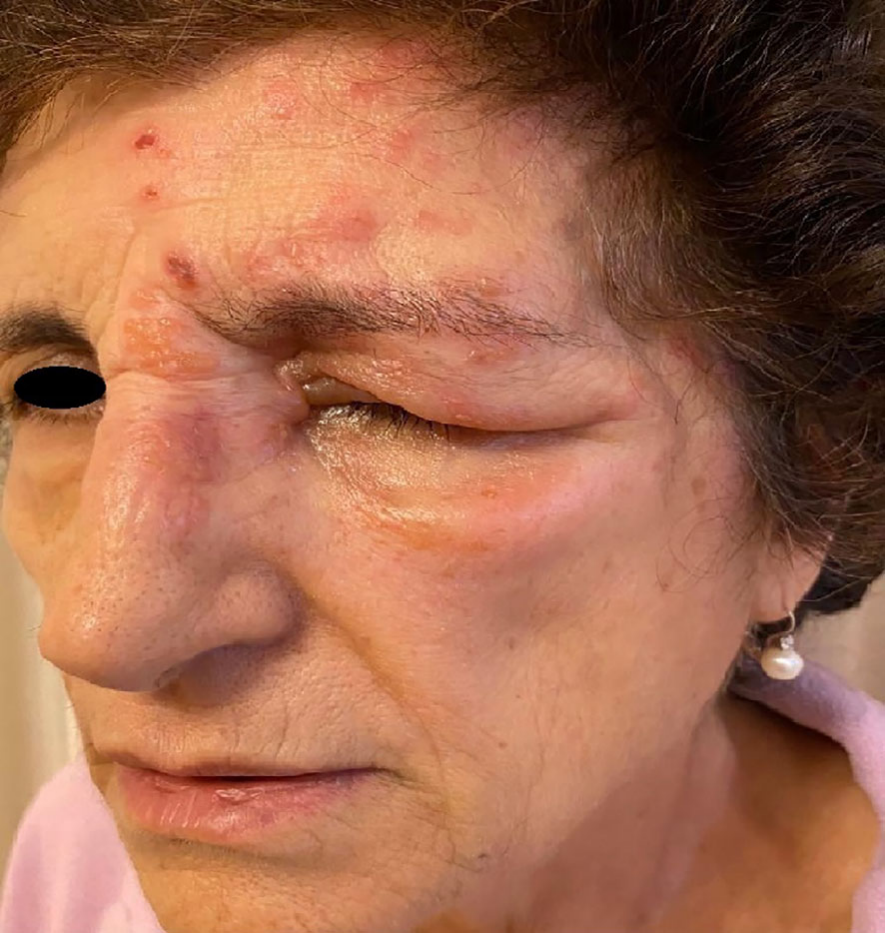
Vescicular eruption along the first and second trigeminal branches (V1 and V2) with dermatomeral distribution

The patient reported painful skin symptoms and visual disturbances. The rash had presented 7 days after the third dose of Pfizer vaccine. On dermatological examination, a vesicular eruption was noted, which was distributed along the first and second trigeminal branches (V1 and V2). In addition, there was diffuse edema of the left eye, which hindered visual acuity, with conjunctival hyperemia. The patient reported that before the rash she had experienced a burning sensation without skin signs the previous day. In addition, she denied having had similar episodes during her lifetime. A diagnosis of HZO was made and systemic antiviral therapy was prescribed, with resolution of the manifestations after 10 days.

Varicella Zoster Virus (VZV) is a DNA virus responsible for chickenpox that remains latent in the involved cranial nerves or root ganglia after initial infection. Immunosuppression, trauma, sunburns, drugs or fever may be a cause of reactivation of the virus.^5^

Elderly individuals are at greater risk of developing HZO, because age‐related immunosenescence is the major risk factor for this condition.^6^ HZO is a disabling condition because it can impair patients' vision.^7^ Corneal lesions and uveitis are the most frequently associated manifestations and can cause blindness if not treated in time.^6^, ^7^

There are few cases of HZO after anti‐SARS‐CoV‐2 vaccine mRNAs in the literature.^7^, ^8^ Although the pathophysiologic mechanism underlying this correlation is still poorly understood, it is hypothesized that the vaccine may slatentize VZV in some patients, particularly in the elderly with an immunosenescent condition.^5^, ^7^ We believe it is important to report our case for the literature, especially because in view of the third dose aimed primarily at immunocompromised patients, the risk of developing adverse reactions is certainly increased. However, given the responsiveness to systemic antiviral treatments, we believe that the risk–benefit ratio is clearly in favor of vaccination and possible side effects should not hinder the global vaccination campaign.

We believe that further studies on this topic are needed to confirm these hypotheses.

## CONFLICT OF INTEREST

G. Fabbrocini acted as a speaker or consultant for Abbvie, Amgen, Eli Lilly, Janssen, Leo‐Pharma, Almyrall, Novartis, and UCB. None of the contributing authors has any conflict of interest, including specific financial interests of relationships and affiliation relevant to the subject matter or discussed materials in the manuscript.

## Data Availability

Data sharing not applicable to this article as no datasets were generated or analysed during the current study.
